# Folded Spectrum VQE: A Quantum Computing Method for the Calculation of Molecular Excited States

**DOI:** 10.1021/acs.jctc.3c01378

**Published:** 2024-03-16

**Authors:** Lila Cadi Tazi, Alex J. W. Thom

**Affiliations:** † École Normale Supérieure Paris-Saclay, Université Paris-Saclay, Gif-sur-Yvette 91190, France; ‡ Yusuf Hamied Department of Chemistry, University of Cambridge, Cambridge CB2 1EW, U.K.

## Abstract

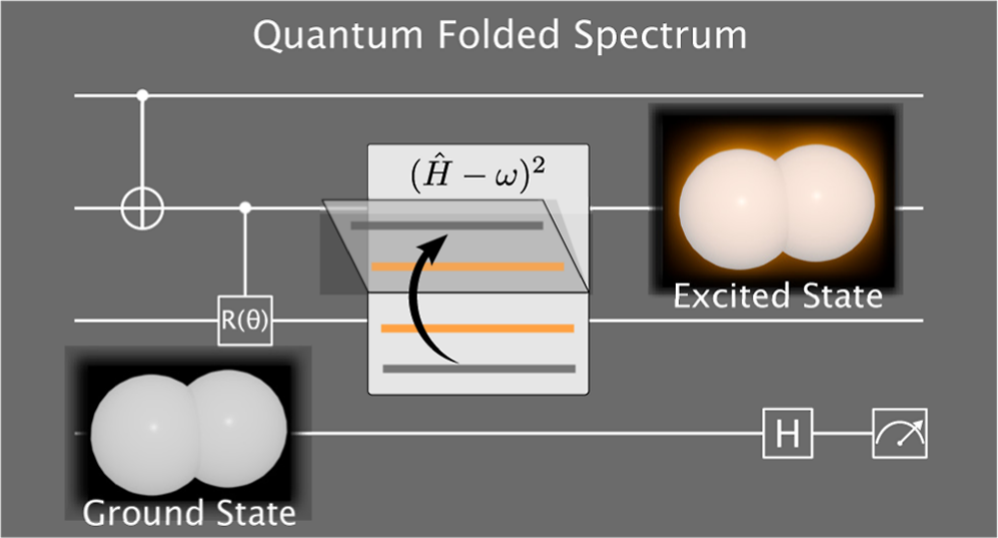

The recent developments of quantum computing present novel potential pathways for quantum chemistry as the scaling of the computational power of quantum computers could be harnessed to naturally encode and solve electronic structure problems. Theoretically exact quantum algorithms for chemistry have been proposed (e.g., quantum phase estimation), but the limited capabilities of current noisy intermediate-scale quantum devices motivated the development of less demanding hybrid algorithms. In this context, the variational quantum eigensolver (VQE) algorithm was successfully introduced as an effective method to compute the ground-state energies of small molecules. This study investigates the folded spectrum (FS) method as an extension of the VQE algorithm for the computation of molecular excited states. It provides the possibility of directly computing excited states around a selected target energy using the same quantum circuit as for the ground-state calculation. Inspired by the variance-based methods from the quantum Monte Carlo literature, the FS method minimizes the energy variance, thus, in principle, requiring a computationally expensive squared Hamiltonian to be applied. We alleviate this potentially poor scaling by employing a Pauli grouping procedure to identify sets of commuting Pauli strings that can be evaluated simultaneously. This allows for a significant reduction in the computational cost. We applied the FS-VQE method to small molecules (H_2_, LiH), obtaining all electronic excited states with chemical accuracy on ideal quantum simulators. Furthermore, we explore the application of quantum error mitigation techniques, demonstrating improved energy accuracy on noisy simulators compared with simulations without mitigation.

## 1 Introduction

Computing the electronic structure of molecules and materials is crucial for the prediction of chemical and structural properties. Theoretical chemists and physicists have acknowledged the essential challenges that must be addressed, but the exponential scaling dimensionality of electronic structure problems seems to be insurmountable on classical computing resources. As a result, the theoretical study of large molecules or materials using ab initio methods such as coupled cluster (CC) is impractical. Hence, less costly methods involving approximations are generally employed at the cost of loss of accuracy and predictive power. The emergence of quantum computing presents potential novel pathways for theoretical chemistry as quantum resources show exponentially scaling computational power that could be harnessed to naturally encode and solve quantum problems. Although exponential speedup may not be achieved, a polynomial acceleration could be ground-breaking for quantum chemistry applications.^1^

In this context, the variational quantum eigensolver (VQE)^2^ was introduced as an effective algorithm to find the lowest eigenvalue of a quantum observable. In particular, it can compute the ground-state energy of a molecular Hamiltonian. The capability of VQE for electronic ground-state computation of small molecules has been extensively studied,^3^ but the effective and direct computation of excited states remains elusive.

In this study, we propose a variant of VQE that aims to compute molecular excited states. It uses the folded spectrum (FS) method to reorder the Hamiltonian’s eigenspectrum, thus allowing for the direct computation of highly excited states. Although this method is documented in the literature, its quantum implementation was considered too costly due to the squared number of terms of the measured operator.^4,5^ Here, we show that a Pauli grouping procedure reduces the required number of measurements, thereby making the cost of the FS method reasonable. The effect of Pauli grouping is particularly significant for second quantized molecular Hamiltonians, as a result of their particular structure. Finally, we present FS-VQE results obtained on a noisy quantum simulator and show the successful use of quantum error mitigation techniques on this algorithm.

## 2 Variational Quantum Eigensolver

**Figure 1:**
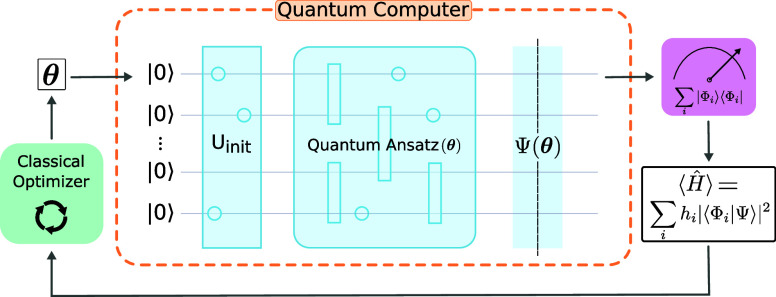
Principle of VQE algorithm.

The VQE^2^ is a hybrid quantum-classical algorithm (see Figure 1). Its purpose is to find the lowest eigenvalue of a given quantum operator. It can be applied to quantum chemistry problems to obtain the electronic ground-state of a molecule, by focusing on the molecular Hamiltonian *Ĥ*.

The algorithm relies on an *ansatz* to prepare a trial electronic wave function on the quantum computer. The ansatz takes the form of a parametric quantum circuit whose parameters, denoted as **θ**, are angles in rotation gates. Details of the ansatz design are given in Section 2.1.

The quantum subroutine prepares a parametric trial wave function Ψ(**θ**) on a qubit register. This quantum state can be assessed by measuring the qubits: from the measurement results, the expectation value of the molecular Hamiltonian  can be computed on a classical computer (see Section 4.1). This value corresponds to the average electronic energy of the trial wave function Ψ(**θ**). A classical optimizer is then used to adjust the parameters **θ** in the ansatz in order to minimize the value of . By means of the variational principle, the minimal expectation value obtained for a set of parameters **θ**_opt_ is an upper bound on the Hamiltonian’s ground energy. The quantum state prepared with the optimal, final angles **θ**_opt_ is a representation of the molecule’s ground-state electronic wave function.

### 2.1 Ansatz

In the context of VQE, the ansatz is a parametric quantum circuit aiming to explore the wave function search space. The ansatz design can take various forms as different properties are targeted.^6^ The number of parameters in the circuit is key to the success of the optimization procedure; a very large number of parameters may lead to intractable optimization.

The so-called *chemically motivated ansatz* class includes ansätze inspired by quantum chemistry methods.^6^ Their advantage is that the prepared states are by design physically relevant (the number of electrons and total spin are conserved). However, they often require a large number of parameters and deep quantum circuits, which limits both the optimization success and their feasibility on the noisy intermediate-scale quantum (NISQ) hardware.

Another approach is to design *hardware motivated ansätze*.^6^ Such ansätze are constructed to be efficiently implemented on quantum computers. Strong constraints in terms of quantum gates, qubit connectivity, number of two-qubit gates, global circuit depth, etc., are defined in accordance with the capability of the target hardware. These ansätze are computationally advantageous, but they do not offer a guarantee on the physical properties of the prepared trial states, thus limiting the convergence.

#### 2.1.1 Unitary Coupled Cluster Ansatz

Unitary coupled cluster (UCC) is a widely used chemically motivated ansatz for electronic wave functions in quantum computing. It is a unitary variant of the well-known CC theory. Like CC, UCC is based on a reference wave function (often Hartree–Fock (HF), and it creates linear combinations of excited determinants using excitation operators T̂
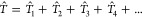
1
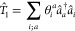
2
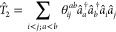
3where  is the operator of all single excitations,  is the operator of all double excitations, etc.  and  are, respectively, the Fermionic creation and annihilation operators acting on orbital *k*. Indices *i*, *j* denote occupied orbitals and *a*, *b* virtual orbitals. Parameters **θ** are optimized to obtain the CC wave function.

Because the CC operator  is not unitary, it cannot be directly implemented on a quantum circuit. To create a unitary variant of CC, the cluster operator needs to be modified to become anti-Hermitian as the exponentiation of an anti-Hermitian operator is unitary
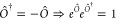
4

Therefore, the anti-Hermitian cluster operator  is considered. The UCC ansatz state is created similar to the CC state
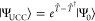
5

For the UCC state to serve as a quantum ansatz, the operator  must be expressed in terms of quantum gates. Since excitation operators do not commute, a Trotterization step is required to decompose the exponentiated operator.^7^ The Trotter decomposition is given by
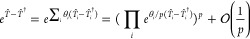
6with  the excitation operators defined in eqs 2 and 3 and *p* is the Trotter decomposition order. In this work, the order *p* = 1 was implemented as it was proven to be an exact and general form of UCC under the condition of an appropriate ordering of excitations^8^ (details in Section 2.1.2). This large form of UCC is truncated to a rank *k* corresponding to the highest excitation considered (for example, UCCSD is *k* = 2: only single and double excitations). The resulting smaller ansatz is therefore
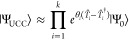
7with parameters **θ** to optimize.

**Figure 2:**
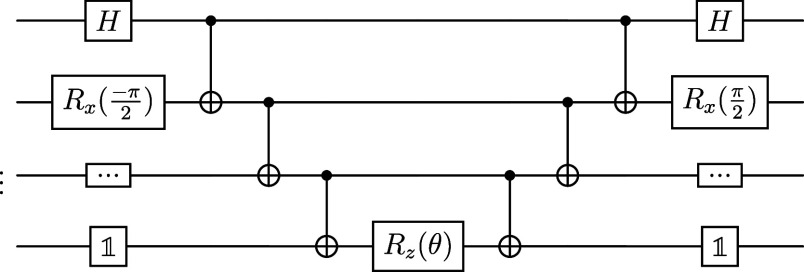
Pauli Gadget circuit for implementing .

The Trotterized and truncated UCC operator can be translated into quantum gates in two steps. The first step is to map the  and â of excitation operators into Pauli strings (tensor products of Pauli matrices, acting on several qubits), as described in Section 2.2. Then, each exponentiated Pauli string can be translated into a Pauli gadget,^9^ as shown in Figure 2. The ansatz parameters **θ** are the rotation angles in the gates *R*_*z*_, denoting single qubit rotations around the *Z*-axis. The sequence of Pauli gadgets gathered into one single circuit constitutes the UCC ansatz.

#### 2.1.2 Ordering of Excitation Operators

The Trotterized form of UCC is a product of non-commuting terms, making the ordering of excitation operators an important hyperparameter of the ansatz. In ref (8), authors have proposed a universal ordering of the excitations allowing us to reach any Fermionic state. Their method is employed in this study. Considering a single determinant reference state |Φ_0_⟩ (chosen as the HF wave function here), we iterate through the occupied indices *j* of |Φ_0_⟩. For each index, all single excitations involving index *j* are added to the ansatz, followed by all double excitations with index *j* and so on for all excitations of higher rank involving orbital *j*. This procedure is repeated for all occupied indices *j* of the reference state, eventually adding all UCC excitations to the circuit.

### 2.2 Fermion-To-Qubit Mapping

Second quantized Fermionic operators can be mapped to qubit operators that can be implemented on a quantum circuit.

Different mapping schemes are available, the most common being the Jordan–Wigner (JW)^10^ and Bravyi–Kitaev (BK)^11^ transformations. In the second quantized formalism, Fermionic operators are expressed as sums of the creation and annihilation operators. Fermion-to-qubit mapping is a systematic formula to translate creation and annihilation operators into Pauli strings. Any second quantized operator
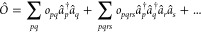
8can be mapped to
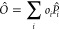
9with  being Pauli strings and *o*_*i*_ scalars.

In this work, JW mapping was employed.

#### 2.2.1 Jordan–Wigner Mapping

In this formalism, each qubit represents a Fermionic state (a spin–orbital for molecules), with the qubit state |0⟩ corresponding to an unoccupied state and |1⟩ to an occupied state. The creation and annihilation operators are mapped using the transformation in eq 10 for a *N*-qubit register corresponding to *N* electronic spin–orbitals.
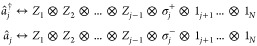
10with gates
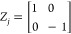

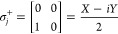

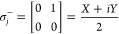
applied to qubit *j*.

The σ^+^ and σ^–^ gates act as qubit creation and annihilation operators, while the *Z* gates are required to conserve the anti-commutation relations
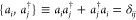
11

### 2.3 VQE Procedure

**Figure 3:**
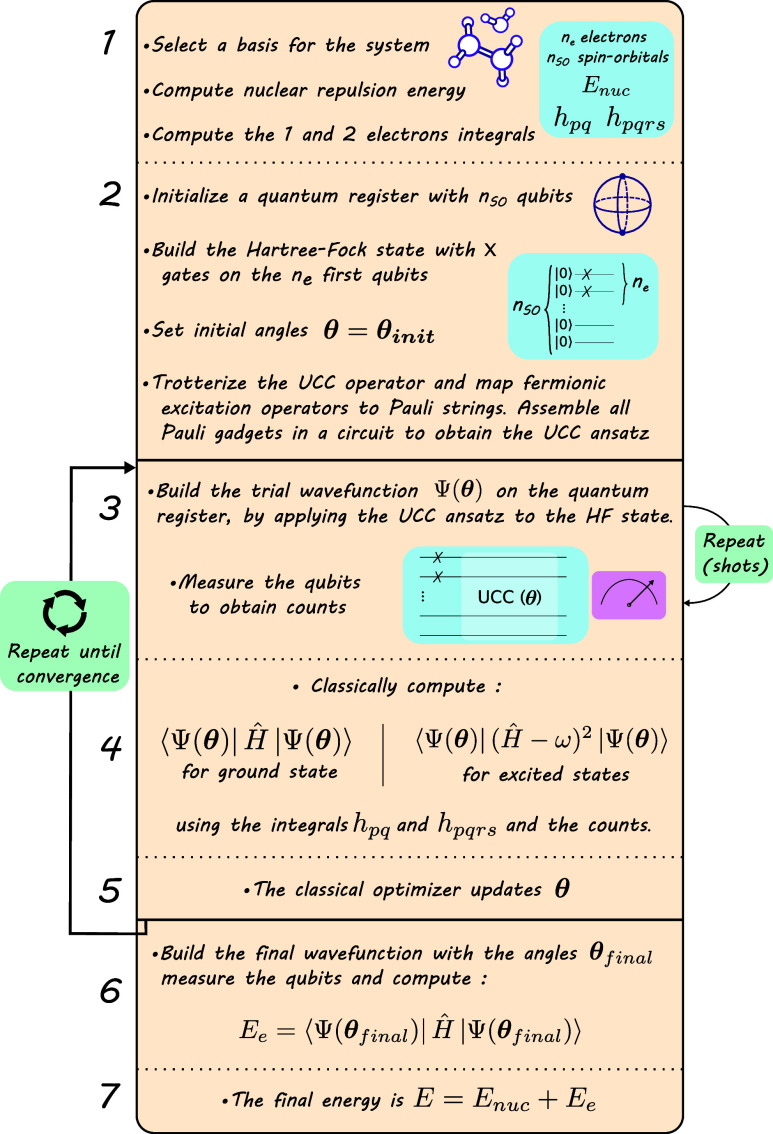
Summary of the preprocessing steps and VQE algorithm to obtain a molecule’s energy.

The procedure implemented in this work is summarized in Figure 3.

## 3 Excited States

The standard VQE algorithm applied to molecular systems allows one to compute the ground-state of the electronic wave function. It is not primarily designed to compute excited states as it relies on the minimization of the average energy. Several approaches have been proposed to reach excited states with quantum algorithms, including quantum subspace expansion (QSE),^12^ variational quantum deflation (VQD)^13^ similar to orthogonally constrained VQE (OC-VQE),^14,15^ witnessing eigenstates (WAVES)^4^ or quantum equation of motion.^16,17^

The FS method has also been reported in the literature,^6^ but the presence of a quadratic term in *Ĥ* is thought to be prohibitive, and it is expected to scale as  relative to the system size.^14^ To the best of our knowledge, no extensive study of this method has been reported.

### 3.1 Folded Spectrum Method

The principle of the FS method is to minimize the expectation value of the FS operator  instead of the Hamiltonian *Ĥ*, with ω an arbitrary target energy.

This method is also known as state-specific variance minimization in the quantum Monte Carlo (QMC) literature, where it has been actively employed and studied for many years.^18−20^

Let |Ψ⟩ be an eigenstate of the Hamiltonian *Ĥ*. It satisfies the time-independent Schrödinger equation
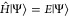
12

The linearity of the Schrödinger equation allows one to write eq 13 for all |Ψ⟩ eigenstates of *Ĥ*, *E* the associated eigenvalues, and ω an arbitrary scalar.
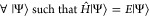

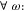

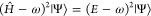
13

**Figure 4:**
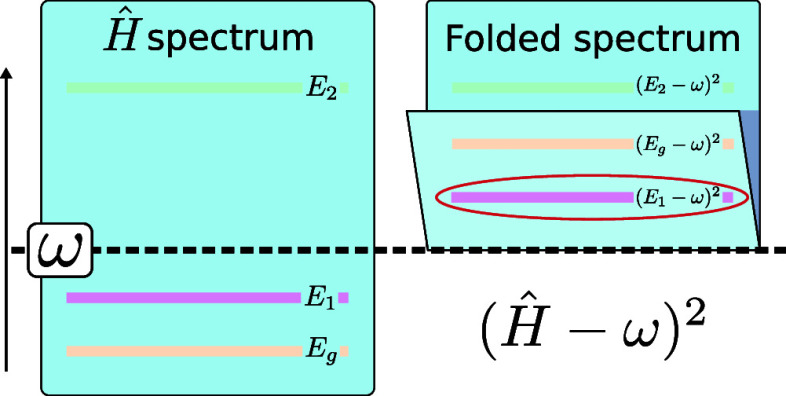
Illustration of the FS method. Colored lines represent eigenstates along a vertically ascending axis. The eigenspectrum of *Ĥ* (left) is *folded* around ω in the spectrum of  (right), causing its eigenvalues to reorder. The lowest eigenvalue of the FS, circled in red, is the excited eigenstate of *Ĥ* originally closest to ω.

The FS operator  and the Hamiltonian *Ĥ* share the same eigenstates but with a reordering in the eigenvalues (corresponding to a *fold* around ω).^21^ The lowest lying eigenstate of the folded operator is the one with an energy *E*_*i*_ closest to ω (see Figure 4).

By minimizing the expectation value of the FS operator , one can find an eigenstate of *Ĥ* such that  is minimal and thus obtain an excited state of the Hamiltonian, close to the target energy ω. In practice, we perform expectation value minimization with a VQE procedure, and the obtained wave function is an approximation of the true eigenstate given by the ansatz. The cost function of interest is
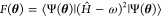
14

All excited states of the Hamiltonian may be obtained by modifying the parameter ω over a wide enough range of energies.

One major limitation of the FS method is that it requires a squared Hamiltonian, containing a large number of Fermionic terms and thus of Pauli strings compared to the Hamiltonian itself. However, by using a Pauli reduction and grouping procedure, as described in Section 4.2, the number of required measurements can be considerably reduced.

## 4 Methods

### 4.1 Computing Expectation Values

The expectation value of quantum operators represented by Pauli strings (as in eq 9) can be decomposed, as shown in eq 15, with  Pauli strings and *o*_*i*_ scalar coefficients.
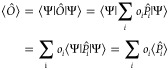
15

The expectation value of Ô can be computed by classically summing the expectation values of each Pauli string  weighted by the precomputed coefficients *o*_*i*_. In molecular Hamiltonians, the coefficients *o*_*i*_ are typically denoted by *h*_*i*_ and are formed through linear combinations of one-body and two-body integrals.

Once the trial state |Ψ⟩ is prepared on the quantum register, we measure the qubits and repeat the preparation and measurement procedure several times (*shots*). Ultimately, we obtain some *counts* that form estimates for |Ψ⟩ populations. Qubit measurements are usually performed in the computational basis denoted , corresponding to the values 0 or 1 for each of the *n* qubits: {Φ_*i*_} = {|0...00⟩, |0...01⟩, ..., |1...11⟩} in the binary order.

In this basis, the spectral decomposition of |Ψ⟩ is
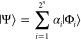
16with
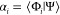
17

Measurable quantities are the populations for each basis vector of the computational basis
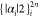
18

Note that the counts provide *estimates* of the populations due to finite sampling. The final precision ϵ in the results is directly correlated with the number of shots taken *s* as  (see Appendix A). Results can be made arbitrarily close to the theoretical value by increasing the number of shots, but this can lead to considerable computing time. To reduce the number of quantum measurements, a Pauli grouping routine can be used, as discussed in the next section.

The derivation of expectation values from the count results is explained below for diagonal and non-diagonal operators.

#### 4.1.1 Diagonal Operators

The expectation value of diagonal Pauli operators in the computational basis (i.e., tensor products of *I* and *Z*) can be directly computed from the counts. Such operators can be decomposed in the computational basis as a sum of projectors
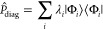
19with λ_*i*_ corresponding to the eigenvalues of the operator, namely, +1 or −1 for products of *I* and *Z* Pauli operators.

The expectation value of  is therefore
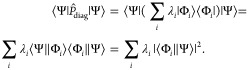
20

Consequently
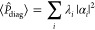
21is directly accessible from the quantum measurement, by classically summing the count results weighted by the eigenvalues of the Pauli operator.

Note that all diagonal Pauli operators can be evaluated from the same counts measurement as the λ_*i*_ coefficients are treated classically.

#### 4.1.2 Nondiagonal Operators

Given a non-diagonal operator *P̂* in the qubit basis, it is always possible to find an appropriate basis change to diagonalize it. *X* and *Y* Pauli matrices are non-diagonal in the computational basis, but they can be diagonalized using the following basis changes
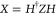
22
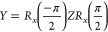
23

Therefore, for a Pauli string *P̂*, it is possible to find a rotated Pauli string *P̃* diagonal in the qubit basis using these basis changes. In general, one can write
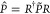
24with
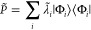
25

The expectation value of *P̂* is then
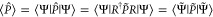
26with
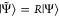
27

Finally
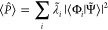
28

To evaluate the expectation value of a non-diagonal Pauli string *P̂*, it is therefore necessary to apply a post-rotation gate *R* to the quantum state |Ψ⟩, which creates a state  in a basis where the Pauli string is diagonal. In practice, the additional post-rotation operator is built with single-qubit gates added after the ansatz. A Hadamard is applied to the qubits where the Pauli operator is *X*, and a  is applied to the qubits where it is *Y*.

The expectation value of a non-diagonal *P̂* is then computed similar to a diagonal Pauli string, using the rotated state  (eq 28).

### 4.2 Pauli String Reduction and Grouping

The number of Pauli strings in the Hamiltonian scales polynomially with the system size and naively, the FS operator can contain up to the square of this number. Evaluating each term one by one can lead to a very large number of measurements, which lowers the potential advantage of the quantum algorithm.

#### 4.2.1 Pauli Reduction

When computing the FS operator , the number of Pauli strings primarily obtained is approximately the square of the number of terms in *Ĥ*. It is possible to simplify and reduce this sum by using the commutation and anticommutation relations between Pauli matrices.^22,23^ In ref (24), authors studied a collection of systems of increasing sizes and concluded that the actual number of Pauli strings in  after Pauli reduction has an effective scaling below  instead of the expected  with *N* the number of spin–orbitals. This result can be extended to our work: the number of terms in the FS operator has a much more favorable scaling with respect to the system size due to Pauli reduction. More formal analyses are required to consolidate this result and assess the feasibility of the FS method for larger systems.

#### 4.2.2 Pauli Grouping

To further reduce the number of quantum evaluation required, one can partition the operators into groups of simultaneously diagonalizable Pauli strings.^25,26^ All the Pauli strings in the same group can have their expectation values determined with a single quantum evaluation, by adding a classical post-processing step. In the formalism of Section 4.1.2, it means that all Pauli strings in the group share the same post-rotation *R* in eq 24.

Formally speaking, a group of operators is simultaneously diagonalizable if and only if the operators commute.^27^ This reduces the problem to identifying groups of commuting Pauli strings in the qubit Hamiltonian or the FS operator. In particular, we want to find a partitioning with a minimal number of groups, leading to a minimal number of quantum evaluations.

Two distinct definitions of commutation can be considered to partition the Pauli strings: qubit-wise commutativity (QWC) or general commutativity (GC).^28^ The former defines that two Pauli strings commute if the Pauli matrices commute at each index. For instance, the group {*IX*, *XX*, *XI*} is QWC since all Pauli matrices for qubit 1 commute and equally for qubit 2.

**Figure 5:**
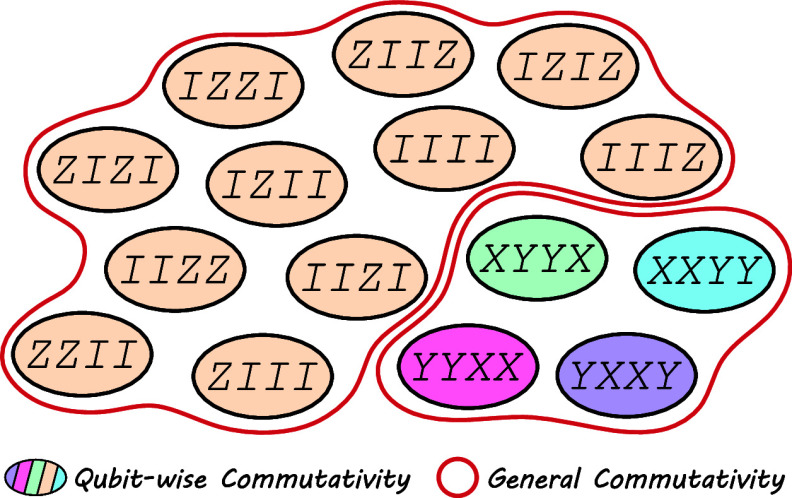
Example of Pauli grouping for the 15 Pauli strings in H_2_ Hamiltonian. Colors show the QWC partitioning that reduces the number of evaluations to 5, and red circles show the GC partitioning that only require 2 evaluations.

General commutativity is fulfilled if the two Pauli strings commute regardless of the single-qubit case. The group {*XX*, *YY*, *ZZ*} is GC although none of the pairs is QWC. The general rule is that each pair must fail to commute at an even number of indices. QWC is, in fact, a special case of GC in which the strings fail to commute at 0 indices. Figure 5 shows an example of QWC and GC partitioning for the electronic Hamiltonian of H_2_.

Finding the optimal Pauli partitioning (in QWC or GC) is equivalent to a graph partitioning problem known as the minimum clique cover problem,^29^ and it is NP hard.^30^ Efficient heuristic algorithms to find a good Pauli partitioning are therefore essential to tend toward scalability for the FS method.^31,32^

**Figure 6:**
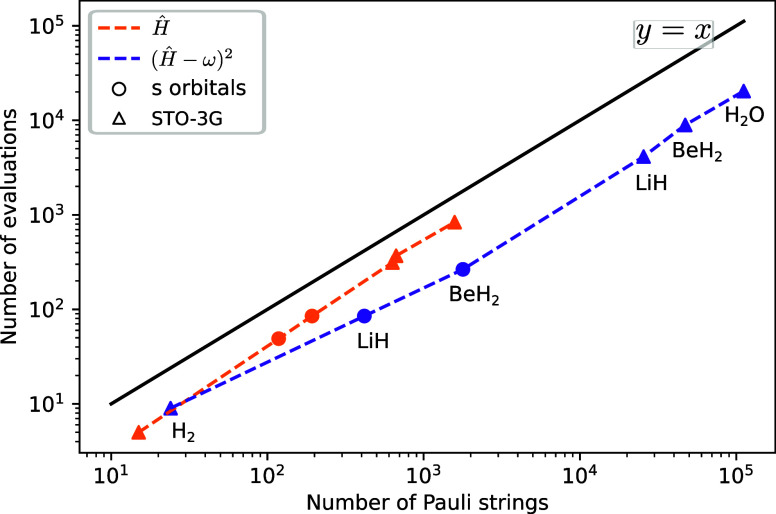
Number of evaluations needed (number of QWC groups) compared to the number of Pauli strings for the Hamiltonian in orange and the FS operator in purple, under JW transformation. Several systems are displayed (labels are in matching order) in STO-3G basis (triangles) or in a minimal basis with only s orbitals (circles). The black line shows the number of evaluations needed if measuring the Pauli strings one by one.

In this work, we use QWC Pauli partitioning as it is more straightforward to implement. We present some results using JW qubit mapping in Figure 6, and Table 2 in Appendix B reports some examples for both J–W and B–K transformations. The number of quantum evaluations is systematically decreased by grouping the Pauli strings. As expected, more evaluations are required for the FS operator compared to those for the Hamiltonian for the same system. Additional results on Hamiltonian grouping for other transformations and systems can be found in refs (31, 33, and 34). As shown in ref (28), GC partitioning is more efficient than QWC, and it would lead to even fewer quantum measurements for the FS method.

#### 4.2.3 Measurement Cost

Pauli grouping for molecular Hamiltonians shows interesting features that may be extended to the FS operator. Electronic Hamiltonians under certain Fermion-to-qubit transformations (such as JW mapping described in Section 2.2) have the convenient property of including a large number of diagonal Pauli strings (with only *I* and *Z* operators). In fact, the product of σ^+^ and σ^–^ operators defined in eq 10 can be decomposed as
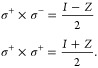
29

Therefore, all terms in the Hamiltonian involving a creation and an annihilation operator for the same spin–orbital are mapped to a diagonal Pauli string in the qubit basis. These terms are the one-body terms with *p* = *q* (*h*_*pp*_) and the two body terms with *p* = *r* and *q* = *s h*_*prpr*_ or *p* = *s* and *q* = *r* (*h*_*prrp*_). For a Hamiltonian describing *n* electrons in *N* spin–orbitals, there are *N* diagonal one-body operators and  diagonal two-body operators. All of these diagonal Pauli strings can therefore be grouped together and evaluated simultaneously.

Additionally, asymptotically dominant terms in the molecular Hamiltonian are two-electrons operators of the form  with *p* ≠ *q* ≠ *r* ≠ *s*.^28^ The number of such terms in a molecular Hamiltonian describing *N* spin–orbitals scales as .

Under JW mapping, two of these terms with disjoint indices, namely,  and  with {*p*, *q*, *r*, *s*}∩{*i*, *j*, *k*, *l*} = ϕ involve disjoint qubits for *X* and *Y* gates, and authors of ref (28). demonstrated that they commute. They showed that using the Baranyai’s graph coloring theorem,^35^ it is possible to partition these  terms into  groups such that the operators within each set have disjoint indices and therefore commute.

In other words, instead of measuring each of the  terms individually, one can perform  measurements only to compute the expectation value of the asymptotically dominant Pauli strings in the molecular Hamiltonian.

Similar to the Hamiltonian, the FS operator can be partitioned into commuting groups to reduce the number of measurements. Here, we present empirical data on the effect of the Pauli grouping for the FS operator. Future studies could aim to establish analytical results on the scaling of the number of evaluations needed for the FS method.

### 4.3 Reference State

When computing the molecular ground-state, the HF determinant can often be used as a reference because of the significant overlap between the HF state and the full configuration interaction (FCI) electronic ground-state. This is not generally true for the excited states. In this case, an excited single determinant or a superposition of two or more determinants having overlap with the target wave function can be used as a reference.

In this study, we have selected relevant references for all electronic excited states by exciting the ground HF determinant with single and double excitations and symmetrizing the spin function when necessary. This procedure can be generalized to larger systems, but we expect that more sophisticated reference states may be required for molecules with strong multireference character. This question remains an essential challenge for the scalability of the FS method. The reference states used in this article are specified in the Results section.

## 5 Noise Robustness and Error Mitigation

The presented algorithm is based on the VQE, which is designed to be amenable to near-term quantum devices. To assess the feasibility of our method on noisy devices, we evaluated its noise robustness by including noise models in our simulations and using zero noise extrapolation (ZNE) and state preparation and measurement (SPAM) error mitigation techniques.^36^

### 5.1 Noise Model

We designed a tuneable and realistic noise model based on the models provided for IBM quantum devices.^37^ This model includes.

- **Gate errors** consisting of a depolarizing channel characterized by one and two qubit gates error rates *p*_1_ and *p*_2_, respectively, followed by thermal relaxation and dephasing processes, driven by *T*_1_ and *T*_2_ characteristic times applied for the gate lengths *t*_gate1_ and *t*_gate2_ for one and two qubits gate.
- **Readout error** characterized by an error probability *p*_SPAM_ for each qubit.

This model can be considered to be realistic for quantum computers, provided that the parameters are adjusted to the calibration data of the real device. However, it excludes noise sources such as state leakage or crosstalk, which are more complex to model. This approximation is widely adopted for its simplicity but may not be suitable for some devices where leakage or cross-talk effects are not negligible.^38^

Parameters *T*_1_, *T*_2_, *t*_gate1_, *t*_gate2_*p*_1_, *p*_2_, and *p*_SPAM_ are provided for IBM machines. Some typical values for the present devices are given in Table 1.^39^

**Table 1:** Values of the Noise Parameters for Different Values of the Scale Factor λ. λ = 1 Corresponds to the Order of Magnitude of the Experimental Values in IBM Machines,^39^ and λ = 0 Is the Ideal Value in a Noiseless Device^a^

λ	*T*_1_ (μs)	*T*_2_ (μs)	*t*_g1_ (ns)	*t*_g2_ (ns)	*p*_1_	*p*_2_	*p*_SPAM_
1	290	145	35	300	1 × 10^–^^4^	1 × 10^–^^3^	1 × 10^–^^2^
0.4	924	461	14	120	4 × 10^–^^5^	4 × 10^–^^4^	4 × 10^–^^3^
0	∞	∞	0	0	0	0	0

a In practice, ideal values were fixed at *T*_1∞_ = 2000 μs and *T*_2∞_ = 1000 μs.

To vary the noise level, we scaled the parameters according to a scale factor λ between their experimental value (λ = 1) and their ideal value (λ = 0). The scaling is performed exponentially for *T*_1_ and *T*_2_ and linearly for all other parameters. Ideal values for *T*_1_ and *T*_2_ were fixed at 2 orders of magnitude above the total length of the circuit, that is, *T*_1∞_ = 2000 μs and *T*_2∞_ = 1000 μs here.

### 5.2 State Preparation and Measurement Mitigation

The SPAM technique aims to mitigate errors introduced during the state preparation and measurement stages.^40,41^ A confusion matrix  is measured by running small calibration circuits on the quantum processor.  represents the noise channel corresponding to the probability that the state preparation or measurement outcome will be incorrect for each qubit. This matrix is then inverted, and the inverse channel  is classically applied to the next experiments as a post-processing step, thus obtaining quasi-probabilities with mitigated SPAM error. The nearest probability distribution is then selected as the mitigated measurement result. This method assumes that the SPAM noise remains constant over multiple experiments close in time on the same device. The calibration circuits should be run regularly to make this assumption reasonable. A more detailed description of the SPAM error channel can be found in ref (38). In our implementation, the confusion matrices are directly extracted from the noise model’s parameters *p*_SPAM_ for each qubit.

### 5.3 Zero Noise Extrapolation

ZNE is a technique that aims to mitigate the effect of noise when evaluating expectation values on quantum processing unit (QPUs).^42^ In ZNE, the hardware noise level is represented by a parameter γ, with γ = 1 corresponding to the actual noise level of the quantum computer, γ > 1 being a noisier hardware, and vice versa.

The principle of ZNE is to intentionally increase the noise level (γ = 3, 5, 7...) and to evaluate the same expectation value *E*_γ_ for the different values of γ. The points obtained are plotted on a *E*_γ_ vs γ curve and fitted with an analytic model. The model provides an extrapolated value at γ = 0 that is retained as the mitigated result *E*_0_, which should be an approximate of the ideal value *E*.

In our implementation, ZNE was employed to mitigate the summed expectation values of each Pauli groups separately. For H_2_, the FS operator contains 24 Pauli strings that can be partitioned using general commutativity into two commuting groups  and 
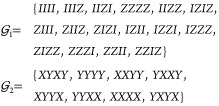
30

For this system, each evaluation of  requires measuring two different circuits, one for each group. A ZNE procedure can be used to mitigate the noise in the summed expectation value of each commuting group.

To increase the noise level γ, we used a method named **unitary folding** that consists of replacing a unitary gate sequence  by a folded version (for example, ). The logical operations of  and its folded versions are the same as , but the effective number of gates in  is three times greater, corresponding to γ = 3. Further folding is performed to implement γ = 5, 7, 9... On noisy devices, folding the circuits effectively corresponds to scaling the gate noise. Thus, this method allows us to artificially implement different values of γ by increasing the circuit depth. This method has the advantage of being very general since it can work for any unitary circuit.

A quadratic model in eq 31 was selected to fit and extrapolate the *E*_γ_(γ) curve.
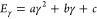
31

ZNE relies on the assumption that folding the circuits corresponds to increasing the noise level by γ overall, which implies that gate noise is the main source of noise. This assumption is not always valid, and, in particular, SPAM error is not scaled with circuit folding, which can make the extrapolation process erroneous. To alleviate this limitation, we employ ZNE mitigation in conjunction with SPAM mitigation such that in theory, SPAM errors are negligible in the ZNE fitted data.

**Figure 7:**
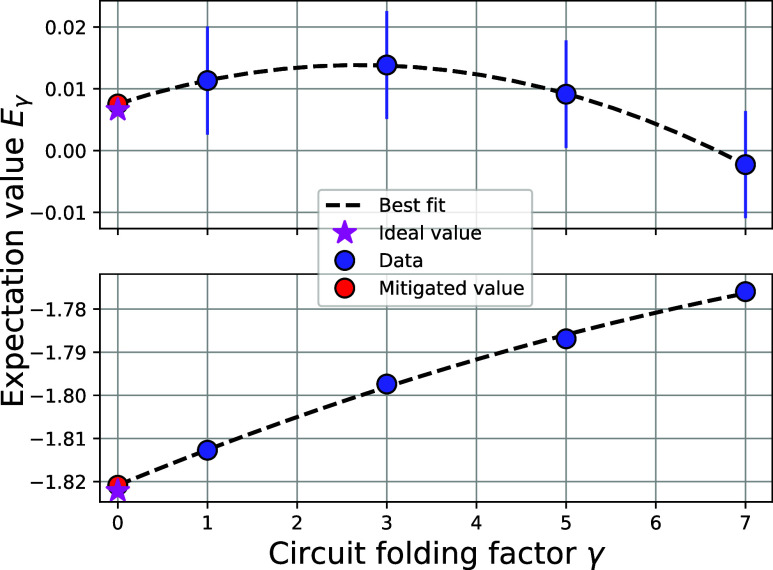
Example of zero noise extrapolation curves coupled with SPAM mitigation on the expectation value of two Pauli commuting groups  (top) and  (bottom). Noise scaling is performed by unitary folding with folding factors 1, 3, 5, and 7, and extrapolation is performed using a quadratic model.

An example of this procedure is given in Figure 7.

## 6 Computational Details

### 6.1 State Tracking

Both the energy and the wave function show continuity along the potential energy surface (PES) of the same electronic state. Therefore, the final energy and final angles for one molecular geometry are good starting points for another close molecular geometry along the PES. We take advantage of this property by setting ω to the previous energy computed on the PES, as well as setting the initial **θ** parameters to the angles found in the previous calculation. In other words, with *k* index representing the evolution along the PES
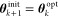
32sets the initial trial wave function for point *k* + 1 as the final wave function of point *k*, and
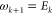
33sets the target energy for the next point to the previous energy computed.

This state tracking method allows one to reduce the optimization time. To ensure continuity of the wave function along the PES, it is necessary to have continuous molecular orbital (MO) coefficients as the geometry changes. The MO coefficients are precomputed for each geometry using the PySCF package.^43^ Phase jumps are possible in the RHF computation as the MO phase can freely change between independent calculations. These phase jumps do not affect the energy, but they do break the continuity in wave function, lowering the effectiveness of state tracking. To avoid phase jumps in the MO coefficients, we compute at each step between close geometries *k* and *k* + 1
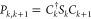
34with *C*_*k*_ the MO coefficient matrix at geometry *k* and *S*_*k*_ the overlap matrix at geometry *k*. The P matrix has a diagonal with ≈ ±1 elements. Negative signs indicate a phase jump between the two geometries. In this case, we rectify the phase of the corresponding MOs in *C*_*k*+1_ matrix and use the rectified MOs in the calculation. This ensures continuity in the ansatz parameters and allows us to facilitate convergence and reduce computation time by employing the state tracking method.

We observed that state and energy tracking, beyond reducing computation time, can also help the optimization convergence for points where it initially fails. When noticing non-converged points on the PES, one can start from a previous converged point and state track toward the desired geometry with smaller geometry steps. This technique usually allows us to obtain better convergence. However, it requires a large number of calculations since the step size needs to be small enough to allow good continuity.

#### 6.1.1 Preventing Jumps between Electronic States

To some extent, state tracking helps prevent jumps between close electronic states as the wave function tends to be continuously evolved along the PES, which is particularly useful when degenerate or quasi-degenerate states are present. However, we observed that jumps still occurred in our computations when the ω parameter was closer to another electronic state for a particular geometry (which is particularly frequent in the presence of large energy gradients or when two electronic states are very close in energy). This behavior is expected for the FS method, but it can be undesirable when trying to follow a particular electronic state on the PES. To prevent jumps, a continuity constraint term can be added to the optimized cost function, ensuring continuity of the parameters along the PES
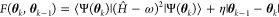
35with η a scaling factor that can be adjusted to balance the relative weights of the two terms in the cost function. This regularization method was used for some isolated points in the PEC, and η was fixed to 0.1 in our implementation, based on empirical trials to find a value that influences optimization toward a region close to the previous calculated point while still maintaining the right landscape and minima.

### 6.2 Classical Optimization

The optimization of variational parameters **θ** is performed using a classical optimizer. The dimensionality of the ansatz and the presence of noise in the cost function make the optimization difficult.

In addition to the finite sampling error that is inherent in quantum computing, NISQ hardware is characterized by the presence of noise within the quantum circuit, making robustness an important feature of quantum algorithms to be applied to current hardware.

Noise-tolerant optimization algorithms are therefore the most appropriate. We use the simultaneous perturbation stochastic approximation (SPSA) optimizer for shot simulations. In addition to being noise tolerant, SPSA also has a constant number of 2 evaluations per iteration that does not scale with the number of parameters.^44^ SPSA uses a stochastic procedure to update the parameters: at each iteration, perturbation Δ**θ** is a randomly generated vector that has a component in every dimension of the optimization problem. The cost function is evaluated at (**θ** + Δ**θ**) and (**θ** – Δ**θ**), and the numerical gradient for each parameter is approximated only from these two measurements. Because the perturbation vector is randomly generated, additional shifts due to noise in the cost function have a minor impact on the optimization process. The noise is, in a sense, absorbed by the stochasticity of the optimizer.

#### 6.2.1 Scaling of Parameters

In the UCC ansatz, the variational parameters are angles in rotation gates, ranging from – π to π. To assist the classical optimizer in finding the optimal angles, we scale the parameters to range from – *c*π to *c*π with *c* being a predetermined constant. This simple procedure helps prevent optimization failures caused by optimization steps being too small to obtain nonzero numerical gradients, especially for shot simulations.

#### 6.2.2 Shot Scheduler

Each evaluation of the cost function *F*_θ_ is performed through a number *s* of measurement procedures, where *s* is named the number of shots. The more shots *s* are taken, the more precise is the measured estimate of the cost function. The precision follows  (see Appendix A).

When measurement accuracy is not crucial, it is advantageous to use a smaller number of shots as a large *s* requires a significant amount of computational time. For this reason, we use an increasing number of shots during the optimization, leading to uncertain measurements far from the optimum where the cost gradients are large and increasing precision when approaching the optimal parameters. For all computations, we used an inverse exponential scheduler of the form
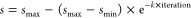
36with *k* > 0 that brings the number of shots from *s*_min_ = 1000 to *s*_max_ = 10,000 with an exponential trend as iterations are performed.

The final measurement after optimization convergence and post-processing of the parameters (see Section 6.3) is performed with 30,000 shots, allowing one to attain a better estimate of the final wave function and energy.

### 6.3 Post-Optimization Processing

#### 6.3.1 Quadratic Fitting

The characteristics of the UCC ansatz search space can be harnessed to improve the parameters **θ**_opt_ found by the optimizer. Let us consider the energy space in the UCCSD formalism, having dim(**θ**) dimensions. In this representation, the parameters **θ**_ideal_ corresponding to eigenstates of the electronic Hamiltonian are located at minima or maxima in the energy space for the relevant excitations. More precisely, the eigenstates are located on the vertices of parabolas in the energy space.

It is possible to take advantage of the particular location of the eigenstates to refine the solutions found by the optimizer. After optimization, one can probe the energy space around each parameter. If the solution found by the optimizer is close to an eigenstate, then the energy space around each **θ** should be a parabola. It is therefore possible to sample a few points around the optimized solution, fit a quadratic equation, and choose the vertex of the fitted parabola as a refined solution. We employ this method as a post-processing step to improve the cost function. The refinement is usually very low (as the optimizer already locates the vertex of parabolas well enough), but in some cases a few tenths of a percent can be gained on the cost function.

#### 6.3.2 Rounding of Parameters

When the electronic wave function is built using the UCC ansatz, it is common for some excitations to be irrelevant because the wave function usually does not contain all possible Slater determinants in the molecule search space. As a result, some parameters of the quantum ansatz have an optimal value of zero and therefore do not participate in the circuit. This can be harnessed to reduce the depth of the ansatz circuit, as described in ref (45). Similarly, some parameters can have an ideal value of π or  when one determinant is completely excited to another, or two determinants have the same contribution to the wave function, respectively. This is particularly common when considering systems with internal symmetries. Here, we take advantage of this feature to improve the accuracy of the optimizer solution by including a rounding post-processing routine. After the optimization (and quadratic fitting, see Section 6.3.1) of the ansatz parameters, we detect close to zero (or close to a fraction of π) parameters and evaluate the cost function when rounding those angles to zero (or to the corresponding fraction of π). If the cost function is improved by rounding them, then the adjusted parameters are maintained. This procedure usually only improves the result very slightly, but the refinement can go up to a few hundredths of a percent in the cost function.

## 7 Results

The FS-VQE method was applied on two small molecules, H_2_ and LiH. The current capability of NISQ hardware (in terms of quantum volume and gate fidelity) is too limited for FS-VQE to obtain reasonable results on real quantum devices. Thus, we restricted our computations to small molecules and small active spaces that are tractable on simulators.

### 7.1 Excited States of H_2_

H_2_ was described with the STO-3G basis including the 1s orbital for each atom, resulting in four spin–orbitals for the system and four qubits after JW transformation.

H_2_ in STO-3G basis is described by two spatial orbitals σ_*g*_ and σ_*u*_ with up and down spin functions. The reference states we used for the three excited states (*T*_1_, *S*_1_ and *S*_2_) are.

- *T*_1_: (σ_*g*_)(σ_*u*_)
- *S*_1_:  +
- *S*_2_: .

**Figure 8:**
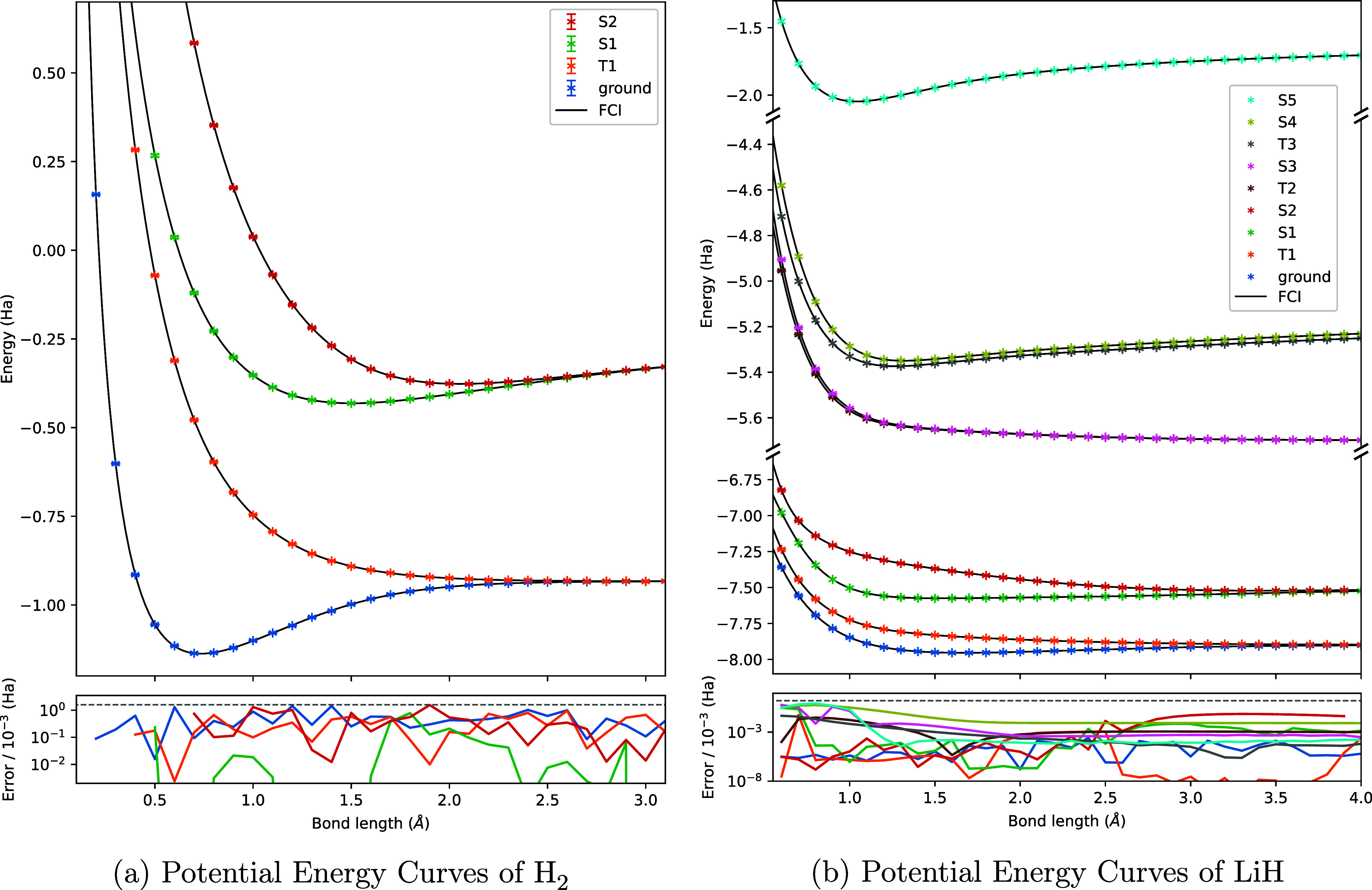
Results of FS-VQE calculations for H_2_ (a) and LiH (b) excited electronic states. Ground-states were obtained with a standard VQE. Colored markers show the (FS)-VQE results, and solid black lines show the FCI energies obtained by numerical diagonalization of the Hamiltonian. The lower subplots represent the **absolute error from FCI**. The error plots were rescaled for clarity, and all nonvisible points are below 10^–3^mHa for H_2_ and below 10^–8^mHa for LiH. (a) Potential energy curves of H_2_ in STO-3G basis obtained on a *shot simulator* (ideal quantum computer with finite sampling *shots* error) using 30,000 shots for the final evaluation. The error bars of finite sampling are represented but smaller than the markers. (b) Potential energy curves of LiH in a minimal s-orbitals-only basis. LiH computations were performed on a *statevector simulator*, ignoring finite sampling error.

The UCCSD circuit for this system of four spin–orbitals and two electrons is composed of three excitation operators that can be implemented in a compiled quantum circuit of depth 71 (with 44 CNOT gates). Computations were performed on Qiskit’s QASM simulator acting like an ideal noiseless quantum computer, including finite sampling error. A shot scheduler between 1000 and 10,000 shots was used during optimization (see Section 6.2.2), and the final measurement was performed with 30,000 shots. Figure 8a shows the results of the FS-VQE algorithm for excited states H_2_ compared to the exact FCI states on the same basis in solid black lines. The FCI energies were obtained by numerically diagonalizing the electronic Hamiltonian matrix to obtain its eigenvalues. The ground-state results were obtained with standard VQE. FS-VQE allows recovering the complete potential energy curves for the three excited states of H_2_ at chemical accuracy. The absolute error is shown in the subplot of Figure 8a.

### 7.2 Excited States of LiH

LiH is a four electron system that can be described with six spin–orbitals in a minimal basis (considering s orbitals only for both atoms). Its excited energies were computed with FS-VQE using six qubits in JW mapping. In this configuration, the UCCSD gate includes eight excitations, resulting in a circuit of depth of 311 with 212 CNOT gates. The computations were performed on Qiskit’s Statevector simulator, allowing the measurement of the qubits’ exact state, thereby avoiding finite sampling error. Note that the results of LiH may differ greatly from the experimental data because the basis set only includes s orbitals, which is a poor approximation for the lithium atom. This minimal description allows us to put the FS-VQE to the test but does not aim to achieve physically accurate results.

Here, LiH is described by three spatial orbitals σ_1_, σ_2_, and σ_3_, each with up and down spin functions. The references we used for each excited state are as follows.

- *T*_1_:
- *S*_1_:  +
- *S*_2_:
- *T*_2_:
- *S*_3_:  +
- *T*_3_:
- *S*_4_:  +
- *S*_5_: .

Figure 8b shows the results of FS-VQE for the potential energy curves of LiH. The solid black lines are the FCI states obtained by diagonalization of the Hamiltonian. Absolute errors compared to FCI are presented in the subplot.

### 7.3 Error Mitigated Results

**Figure 9:**
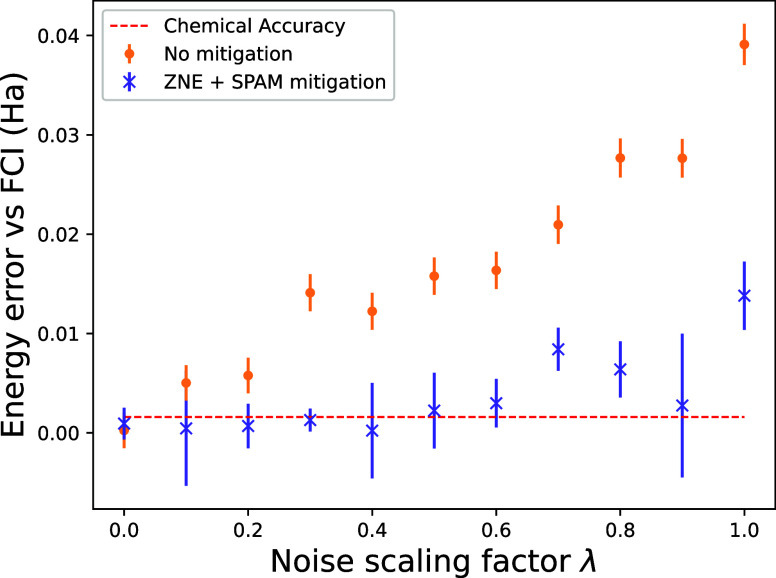
Comparison of the final energy accuracy obtained with non-mitigated and mitigated FS-VQE on the *S*_2_ state of H_2_ at 0.74 Å. Horizontal axis is the simulated level of noise.

The mitigated FS-VQE algorithm was used to compute the highest excited state *S*_2_ of the H_2_ molecule in the STO-3G basis, at a fixed bond length of 0.74 Å, while adjusting the level of noise with the scaling factor λ as described in Section 5.2. 20,000 shots were taken for each circuit evaluation. Figure 9 compares the results of noisy FS-VQE simulations without mitigation with the corresponding results obtained with the combined SPAM and ZNE mitigation methods.

The accuracy of the excited *S*_2_ state energy computation is improved by employing mitigation techniques. When using mitigation, the energy computation reaches chemical accuracy compared to FCI for λ < 0.4, while only λ = 0 is below 1 kcal/mol without mitigation. The noise parameters for λ = 0.4 are given in Table 1.

This may indicate that error mitigation techniques will be useful tools throughout the early fault-tolerant era of QPUs to extract more accurate data from noisy quantum devices. Our results suggest that chemical accuracy for small systems using our algorithm and the described mitigation methods could be reached for quantum devices with improved performances of about an order of magnitude compared with those of the present machines. In the long term, we expect noise mitigation methods to be one of the tools in the error correction arsenal for the early FT era, but logical qubit-based error correction techniques will inevitably be required for large-scale quantum computing.

These experiments are a proof of concept that the FS-VQE algorithm can be combined with mitigation techniques to deal with noise in quantum computations. More detailed and extensive analysis of the use of mitigation techniques in FS-VQE is left for further studies.

## 8 Discussion

### 8.1 Accuracy

In principle, the FS-VQE method with the UCCSD ansatz allows one to recover CC accuracy provided that the optimization converges. For small systems such as H_2_ or LiH with frozen core, CCSD is complete, and in theory we can recover FCI energies in the selected basis. However, for larger systems with more orbitals, single and double excitations are not generally sufficient to reach FCI accuracy, and CC accuracy is expected when implementing UCCSD ansatz. Larger simulations or experiments would be needed for confirmation as our work is restricted to very small systems.

In general, our simulations achieve good accuracy on a simulated noiseless quantum computer, and all points in Figure 8 have an error within the range of chemical accuracy (1 kcal/mol) compared to FCI, as evidenced by the subplots. However, a wide range of errors (between 1.5 and 10^–16^ mHa) and different patterns of error curves are observed. These differences can be attributed to the optimization process: the optimization terminates when a threshold value (10^–9^ in our implementation) is reached in the cost function gradient, leading to various stages of convergence between different runs.

When noise models are included in the simulations, the addition of mitigation techniques is required to reach a chemical accuracy. A simple implementation of SPAM mitigation and zero-noise extrapolation is sufficient to significantly improve the results of FS-VQE, as evidenced in Figure 9. This result is promising for the next early fault-tolerant quantum era with lower error rates, where error mitigation is expected to play a major role and where FS-VQE could produce useful mitigated results.

### 8.2 Scaling and Cost

One asset of the FS-VQE method is that the same circuit can be used for ground-state and excited-state calculations. Excited states only require the evaluation of additional Pauli operators, meaning additional measurement procedures using the same hardware requirements and the same quantum circuit structures (with possibly varying post-rotation gates, representing minor changes).

The FS-VQE method has the disadvantage of involving the Hamiltonian square, making the number of Pauli string expectation values to evaluate larger than that in standard VQE. After a Pauli grouping procedure (as described in Section 4.2), the resulting number of required measurements is greatly reduced, as shown in Figure 6.

Many questions remain open about the feasibility of the FS-VQE algorithm (and variational quantum algorithms in general) for large systems as the classical part of the hybrid algorithm potentially retains intractable stages for large systems. Among these may be mentioned the number of measurements needed, the classical storage of measurement results, or the precomputation of the qubit Hamiltonian and of the FS operator. In particular, the Pauli reduction of the FS operator is a difficult classical task that would need further investigation to become scalable. The Pauli grouping procedure is also a crucial challenge as it was shown to be a NP-hard problem,^28^ while the feasibility of the quantum FS method is highly dependent on it. In addition, optimizing variational parameters becomes more and more challenging as the system size increases, especially due to the Barren plateaux. A careful design of the ansatz can overcome some of these challenges.^46^

The scaling analysis of the proposed algorithm can be divided in terms of the number of circuit evaluations required, the circuit depth, and finally, the number of shots to reach a target accuracy. The number of circuit evaluations corresponds to the number of Pauli groups, which scale as  (discussed in Section 4.2) with respect to system size. The circuit depth depends on the ansatz. In this work, we implement the UCCSD ansatz that has a  scaling in circuit depth, corresponding to the number of double excitations in the cluster operator. However, several ansätze have been proposed in the literature^47,48^ that show a more favorable scaling, such as , while maintaining the same accuracy as UCCSD. Finally, the number of shots needed to achieve a certain accuracy ϵ scales as  (see Appendix A) for each Pauli string. Several techniques have been developed to address the measurement problem in VQAs, including Pauli grouping, measurement weighting, or shadow tomography.^3^

The FS method is a general minimization procedure that can be implemented within algorithms other than VQE to find excited states. Pauli operators are the building blocks of gate-based quantum computing, and we expect Pauli grouping procedures to remain relevant beyond variational algorithms, in which case spectrum folding could be one advantageous method, beyond variational algorithms, to compute molecular excited states on quantum computers.

Employing mitigation techniques seems to be central to obtaining meaningful results on noisy devices, but it comes at the cost of running deeper circuits with more shots. Our implementation of ZNE requires four times more shots compared to the non-mitigated algorithm, for circuits of depth multiplied by γ = 1,3,5,7. This overhead is non-negligible and needs to be addressed in the future.

## 9 Conclusions and Prospects

In this work, we demonstrate that the FS method is a successful approach for computing excited states using the VQE algorithm. The concept of evaluating the FS operator instead of the Hamiltonian to reach excited states is a well-known technique in QMC, and it could also be extended to algorithms other than the VQE in quantum computing. Moreover, the FS procedure is agnostic to the quantum ansatz and the Fermion-to-qubit mapping scheme, and future improvements at any stage of the VQE algorithm can directly benefit this method.

FS allows one to directly compute any excited state around a target energy, which is a considerable asset compared with other methods where excited states are computed sequentially. This advantage is especially important for studying larger systems that have an increasing number of electronic states. It can be particularly useful for the computation of highly excited electronic energies and of great interest for the study of photochemical processes and light–matter interactions.

A major challenge to enable the scaling of variational methods is the preparation of good reference states for larger systems, which is particularly challenging for multireference states. As explained in ref (1), a severe limitation to the scalability of ground-state VQE is the exponentially vanishing overlap of local reference states with the FCI ground-state. Our method also faces this issue, and further research is needed to allow for a systematic and scalable determination of reference states. However, we believe that finding excited states could be a less difficult task if the approach is to compute any excited state around a target energy rather than to search for a specific state (as is the case for ground-state computations). In this scenario, our algorithm could, in principle, find a local minimum in the FS landscape that would correspond to an excited eigenstate. The increasing density of states in larger systems would benefit this approach, and the reference state would thus play a less crucial role.

The main limitation of the FS method is the need to evaluate the squared Hamiltonian. In the quantum computing formalism of the Fermion-to-qubit mapping, this disadvantage can be alleviated by partitioning Pauli operators into commuting groups that can be evaluated simultaneously. The resulting number of evaluations needed to compute the FS operator expectation value is substantially reduced by the Pauli grouping. Despite this improvement, the number of shots required by the FS-VQE method for large systems is still prohibitive on quantum hardware, and further progress is needed to make the method scalable for practical applications. This is particularly central when dealing with noisy quantum processors as error mitigation or error correction techniques are key to obtaining meaningful results but come at the cost of additional quantum resources both in number of shots and number of qubits.

## Appendix A

### Measurement Precision

Measurement precision is directly related to the number of shots taken s. Let us consider the evaluation of  with spectral decomposition

1.1

The measurement of  relies on many repetitions of preparation of and measurement to evaluate the populations |⟨Φ_*i*_⟩Ψ|^2^. Let *V* =  be the target value of the process. can be interpreted as the expected value of a random variable X having possible outcomes , with probabilities given by the Born rule

1.2

Each shot of the experiment is a measure of *X*. By taking s shots, we obtain a set of results *X*_1_,...,*X*_*s*_. Thus

1.3

is approximated by

1.4

In this formalism, Chebyshev’s inequality states that

1.5

with ϵ the precision of the result and σ^2^ the variance of X, making  the variance estimate of the sample by means of the central limit theorem.

σ^2^ can be bounded by a constant,^49^ so it can be deduced that in a worst-case scenario

1.6

Consequently, for a number of shots s, the precision on the expectation value  is of the order of . The extraction of classical information from a quantum system is therefore limited by a finite number of shots. This result is a direct consequence of the probabilistic nature of quantum mechanics.

## Appendix B

### Effectiveness of Pauli Grouping on Some Examples

Some examples for both J–W and B–K transformations are given in Table 2.

**Table 2:** Number of Pauli Strings in the Hamiltonians and FS Operators of Several Molecules in the STO-3G Basis or in a Minimal Basis with s Orbitals Only, Compared with the Number of Groups after Qubit-Wise Commutativity (QWC) Partitioning for Jordan–Wigner (JW) and Bravyi–Kitaev (BK) Transformations

molecule	*e*^–^	basis	qubits	*Ĥ*			(*Ĥ*−ω)^2^		
				Paulis	JW	BK	Paulis	JW	BK
H_2_	2	STO-3G	4	15	5	2	24	9	3
LiH	4	s only	6	118	29	38	417	65	88
LiH	4	STO-3G	12	631	136	211	25,542	2216	3460
BeH_2_	6	s only	8	193	43	46	1783	224	139
BeH_2_	6	STO-3G	14	666	369	324	47,171	8933	8325
H_2_O	10	STO-3G	14	1578	837	746	111,615	20,393	19,596

## References

[ref1] Lee S.; Lee J.; Zhai H.; Tong Y.; Dalzell A. M.; Kumar A.; Helms P.; Gray J.; Cui Z.-H.; Liu W.; et al. Evaluating the evidence for exponential quantum advantage in ground-state quantum chemistry. Nat. Commun. 2023, 14, 1952 10.1038/s41467-023-37587-6. 37029105 PMC10082187

[ref2] Peruzzo A.; McClean J.; Shadbolt P.; Yung M.-H.; Zhou X.-Q.; Love P. J.; Aspuru-Guzik A.; O’Brien J. L. A variational eigenvalue solver on a photonic quantum processor. Nat. Commun. 2014, 5, 4213 10.1038/ncomms5213. 25055053 PMC4124861

[ref3] Tilly J.; Chen H.; Cao S.; Picozzi D.; Setia K.; Li Y.; Grant E.; Wossnig L.; Rungger I.; Booth G. H.; et al. The Variational Quantum Eigensolver: A review of methods and best practices. Phys. Rep. 2022, 986, 1–128. 10.1016/j.physrep.2022.08.003.

[ref4] Santagati R.; Wang J.; Gentile A. A.; Paesani S.; Wiebe N.; McClean J. R.; Morley-Short S.; Shadbolt P. J.; Bonneau D.; Silverstone J. W.; et al. Witnessing eigenstates for quantum simulation of Hamiltonian spectra. Sci. Adv. 2018, 4, eaap9646 10.1126/sciadv.aap9646. 29387796 PMC5787384

[ref5] Zhang F.; Gomes N.; Yao Y.; Orth P. P.; Iadecola T. Adaptive variational quantum eigensolvers for highly excited states. Phys. Rev. B 2021, 104, 075159 10.1103/PhysRevB.104.075159.

[ref6] Cao Y.; Romero J.; Olson J. P.; Degroote M.; Johnson P. D.; Kieferová M.; Kivlichan I. D.; Menke T.; Peropadre B.; Sawaya N. P. D.; et al. Quantum Chemistry in the Age of Quantum Computing. Chem. Rev. 2019, 119, 10856–10915. 10.1021/acs.chemrev.8b00803. 31469277

[ref7] Anand A.; Schleich P.; Alperin-Lea S.; Jensen P. W. K.; Sim S.; Díaz-Tinoco M.; Kottmann J. S.; Degroote M.; Izmaylov A. F.; Aspuru-Guzik A. A quantum computing view on unitary coupled cluster theory. Chem. Soc. Rev. 2022, 51, 1659–1684. 10.1039/d1cs00932j. 35166276

[ref8] Evangelista F. A.; Chan G. K.-L.; Scuseria G. E. Exact parameterization of fermionic wave functions via unitary coupled cluster theory. J. Chem. Phys. 2019, 151, 244112 10.1063/1.5133059. 31893918

[ref9] Nielsen M. A.; Chuang I. L. Quantum Computation and Quantum Information: 10th Anniversary ed.; Cambridge University Press, 2010.

[ref10] Jordan P.; Wigner E. Über das Paulische Äquivalenzverbot. Z. Phys. 1928, 47, 631–651. 10.1007/bf01331938.

[ref11] Bravyi S. B.; Kitaev A. Y. Fermionic Quantum Computation. Ann. Phys. 2002, 298, 210–226. 10.1006/aphy.2002.6254.

[ref12] McClean J. R.; Kimchi-Schwartz M. E.; Carter J.; de Jong W. A. Hybrid quantum-classical hierarchy for mitigation of decoherence and determination of excited states. Phys. Rev. A 2017, 95, 042308 10.1103/physreva.95.042308.

[ref13] Higgott O.; Wang D.; Brierley S. Variational Quantum Computation of Excited States. Quantum 2019, 3, 156 10.22331/q-2019-07-01-156.

[ref14] Lee J.; Huggins W. J.; Head-Gordon M.; Whaley K. B. Generalized Unitary Coupled Cluster Wave functions for Quantum Computation. J. Chem. Theory Comput. 2019, 15, 311–324. 10.1021/acs.jctc.8b01004. 30485748

[ref15] Jones T.; Endo S.; McArdle S.; Yuan X.; Benjamin S. C. Variational quantum algorithms for discovering Hamiltonian spectra. Phys. Rev. A 2019, 99, 062304 10.1103/physreva.99.062304.

[ref16] Ollitrault P. J.; Kandala A.; Chen C.-F.; Barkoutsos P. K.; Mezzacapo A.; Pistoia M.; Sheldon S.; Woerner S.; Gambetta J. M.; Tavernelli I. Quantum equation of motion for computing molecular excitation energies on a noisy quantum processor. Phys. Rev. Res. 2020, 2, 043140 10.1103/physrevresearch.2.043140.

[ref17] Asthana A.; Kumar A.; Abraham V.; Grimsley H.; Zhang Y.; Cincio L.; Tretiak S.; Dub P. A.; Economou S. E.; Barnes E.; et al. Quantum self-consistent equation-of-motion method for computing molecular excitation energies, ionization potentials, and electron affinities on a quantum computer. Chem. Sci. 2023, 14, 2405–2418. 10.1039/D2SC05371C. 36873839 PMC9977410

[ref18] Umrigar C. J.; Wilson K. G.; Wilkins J. W. Optimized trial wave functions for quantum Monte Carlo calculations. Phys. Rev. Lett. 1988, 60, 1719–1722. 10.1103/PhysRevLett.60.1719. 10038122

[ref19] Hanscam R.; Neuscamman E. Applying Generalized Variational Principles to Excited-State-Specific Complete Active Space Self-consistent Field Theory. J. Chem. Theory Comput. 2022, 18, 6608–6621. 10.1021/acs.jctc.2c00639. 36215108

[ref20] Otis L.; Neuscamman E. A promising intersection of excited-state-specific methods from quantum chemistry and quantum Monte Carlo. Wiley Interdiscip. Rev. Comput. Mol. Sci. 2023, 13, e1659 10.1002/wcms.1659.

[ref21] Wang L.-W.; Zunger A. Solving Schrödinger’s equation around a desired energy: Application to silicon quantum dots. J. Chem. Phys. 1994, 100, 2394–2397. 10.1063/1.466486.

[ref22] Aulicino J. C.; Keen T.; Peng B. State preparation and evolution in quantum computing: A perspective from Hamiltonian moments. Int. J. Quantum Chem. 2021, 122, 122 10.1002/qua.26853.

[ref23] Claudino D.; Peng B.; Bauman N. P.; Kowalski K.; Humble T. S. Improving the accuracy and efficiency of quantum connected moments expansions. Quantum Sci. Technol. 2021, 6, 034012 10.1088/2058-9565/ac0292.

[ref24] Suchsland P.; Tacchino F.; Fischer M. H.; Neupert T.; Barkoutsos P. K.; Tavernelli I. Algorithmic Error Mitigation Scheme for Current Quantum Processors. Quantum 2021, 5, 492 10.22331/q-2021-07-01-492.

[ref25] Kandala A.; Mezzacapo A.; Temme K.; Takita M.; Brink M.; Chow J. M.; Gambetta J. M. Hardware-efficient variational quantum eigensolver for small molecules and quantum magnets. Nature 2017, 549, 242–246. 10.1038/nature23879. 28905916

[ref26] McClean J. R.; Romero J.; Babbush R.; Aspuru-Guzik A. The theory of variational hybrid quantum-classical algorithms. New J. Phys. 2016, 18, 023023 10.1088/1367-2630/18/2/023023.

[ref27] Horn R. A.; Johnson C. R. Matrix Analysis; Cambridge University Press: Cambridge, 2012, pp 425–516.

[ref28] Gokhale P.; Angiuli O.; Ding Y.; Gui K.; Tomesh T.; Suchara M.; Martonosi M.; Chong F. T. $O(N̂3)$ Measurement Cost for Variational Quantum Eigensolver on Molecular Hamiltonians. IEEE Trans. Quantum Eng. 2020, 1, 1–24. 10.1109/TQE.2020.3035814.

[ref29] Golumbic M. C. Algorithmic graph theory and perfect graphs. In Annals of Discrete Mathematics, 2nd ed.; North-Holland, 2004.

[ref30] Miller R., Ed. In Complexity of computer computations; The IBM Research Symposia Series; Springer: New York, NY, 1972, pp 85–103.

[ref31] Verteletskyi V.; Yen T.-C.; Izmaylov A. F. Measurement optimization in the variational quantum eigensolver using a minimum clique cover. J. Chem. Phys. 2020, 152, 124114 10.1063/1.5141458. 32241154

[ref32] Huggins W. J.; McClean J. R.; Rubin N. C.; Jiang Z.; Wiebe N.; Whaley K. B.; Babbush R. Efficient and noise resilient measurements for quantum chemistry on near-term quantum computers. Npj Quantum Inf. 2021, 7, 23 10.1038/s41534-020-00341-7.

[ref33] Izmaylov A. F.; Yen T.-C.; Lang R. A.; Verteletskyi V. Unitary Partitioning Approach to the Measurement Problem in the Variational Quantum Eigensolver Method. J. Chem. Theory Comput. 2020, 16, 190–195. 10.1021/acs.jctc.9b00791. 31747266

[ref34] Yen T.-C.; Ganeshram A.; Izmaylov A. F. Deterministic improvements of quantum measurements with grouping of compatible operators, non-local transformations, and covariance estimates. Npj Quantum Inf. 2023, 9, 14. 10.1038/s41534-023-00683-y PMC11041696 38665255

[ref35] Baranyai Z. On the factorization of the complete uniform hypergraphs. In infinite and Finite Sets; , 1974, pp 91–108.

[ref36] Cai Z.; Babbush R.; Benjamin S. C.; Endo S.; Huggins W. J.; Li Y.; McClean J. R.; O’Brien T. E. Quantum error mitigation. Rev. Mod. Phys. 2023, 95, 045005 10.1103/RevModPhys.95.045005.

[ref37] Qiskit contributors. Qiskit: An Open-Source Framework for Quantum Computing, 2023.

[ref38] Georgopoulos K.; Emary C.; Zuliani P. Modeling and simulating the noisy behavior of near-term quantum computers. Phys. Rev. A 2021, 104, 062432.

[ref39] IBM Quantum — quantum-computing.ibm.Com. https://quantum-computing.ibm.com/, 2023.

[ref40] Bravyi S.; Sheldon S.; Kandala A.; Mckay D. C.; Gambetta J. M. Mitigating measurement errors in multiqubit experiments. Phys. Rev. A 2021, 103, 042605 10.1103/PhysRevA.103.042605.

[ref41] Nation P. D.; Kang H.; Sundaresan N.; Gambetta J. M. Scalable Mitigation of Measurement Errors on Quantum Computers. PRX Quantum 2021, 2, 040326 10.1103/PRXQuantum.2.040326.

[ref42] Giurgica-Tiron T.; Hindy Y.; LaRose R.; Mari A.; Zeng W. J. Digital zero noise extrapolation for quantum error mitigation. In IEEE International Conference on Quantum Computing and Engineering (QCE), 2020.

[ref43] Sun Q.; Berkelbach T. C.; Blunt N. S.; Booth G. H.; Guo S.; Li Z.; Liu J.; McClain J. D.; Sayfutyarova E. R.; Sharma S.; et al. PySCF: the Python-based simulations of chemistry framework. Wiley Interdiscip. Rev. Comput. Mol. Sci. 2017, 8, e1340 10.1002/wcms.1340.

[ref44] Bhatnagar S.; Prasad H.; Prashanth L. Stochastic Recursive Algorithms for Optimization; Springer London, 2013, pp 41–76.

[ref45] Filip M.-A.; Fitzpatrick N.; Ramo D. M.; Thom A. J. W. Reducing unitary coupled cluster circuit depth by classical stochastic amplitude prescreening. Phys. Rev. Res. 2022, 4, 023243 10.1103/physrevresearch.4.023243.

[ref46] Grimsley H. R.; Barron G. S.; Barnes E.; Economou S. E.; Mayhall N. J. Adaptive, problem-tailored variational quantum eigensolver mitigates rough parameter landscapes and barren plateaus. Npj Quantum Inf. 2023, 9, 19 10.1038/s41534-023-00681-0.

[ref47] Magoulas I.; Evangelista F. A. Linear-Scaling Quantum Circuits for Computational Chemistry. J. Chem. Theory Comput. 2023, 19, 4815–4821. 10.1021/acs.jctc.3c00376. 37410884 PMC10413858

[ref48] Burton H. G. A. Accurate and gate-efficient quantum ansätze for electronic states without adaptive optimization, 2023. arXiv:2312.09761 [physics.chem-ph]. https://arxiv.org/abs/2312.09761v3 (accessed 19 Jan 2024).

[ref49] McClean J. R.; Babbush R.; Love P. J.; Aspuru-Guzik A. Exploiting Locality in Quantum Computation for Quantum Chemistry. J. Phys. Chem. Lett. 2014, 5, 4368–4380. 10.1021/jz501649m. 26273989

